# Arsenic Trioxide Modulates DNA Synthesis and Apoptosis in Lung Carcinoma Cells

**DOI:** 10.3390/ijerph7051996

**Published:** 2010-04-28

**Authors:** Alice M. Walker, Jacqueline J. Stevens, Kenneth Ndebele, Paul B. Tchounwou

**Affiliations:** 1 Molecular Toxicology Research Laboratory, NIH RCMI-Center for Environmental Health, College of Science, Engineering and Technology, Jackson State University, 1400 JR Lynch Street, Box 18540, Jackson, MS 39217, USA; E-Mail: alice.m.walker@jsums.edu; 2 Molecular and Cellular Biology Research Laboratory, NIH RCMI-Center for Environmental Health, College of Science, Engineering and Technology, Jackson State University, 1400 JR Lynch Street, Box 18540, Jackson, MS 39217, USA; E-Mail: jacqueline.j.stevens@jsums.edu; 3 Laboratory of Cancer Immunology: Target Identification and Validation, College of Science, Engineering and Technology, Jackson State University, 1400 JR Lynch Street, Box 18540, Jackson, MS 39217, USA; E-Mail: kenneth.ndebele@jsums.edu

**Keywords:** arsenic trioxide (As_2_O_3_), [^3^H]thymidine incorporation, apoptosis, cell cycle regulation, p38 MAP kinase, caspase-3 fluorescein isothiocyanate (FITC)

## Abstract

Arsenic trioxide, the trade name Trisenox, is a drug used to treat acute promyleocytic leukemia (APL). Studies have demonstrated that arsenic trioxide slows cancer cells growth. Although arsenic influences numerous signal-transduction pathways, cell-cycle progression, and/or apoptosis, its apoptotic mechanisms are complex and not entirely delineated. The primary objective of this research was to evaluate the effects of arsenic trioxide on DNA synthesis and to determine whether arsenic-induced apoptosis is mediated via caspase activation, p38 mitogen–activated protein kinase (MAPK), and cell cycle arrest. To achieve this goal, lung cancer cells (A549) were exposed to various concentrations (0, 2, 4, 6, 8, and 10 μg/mL) of arsenic trioxide for 48 h. The effect of arsenic trioxide on DNA synthesis was determined by the [^3^H]thymidine incorporation assay. Apoptosis was determined by the caspase-3 fluorescein isothiocyanate (FITC) assay, p38 MAP kinase activity was determined by an immunoblot assay, and cell-cycle analysis was evaluated by the propidium iodide assay. The [^3^H]thymidine-incorporation assay revealed a dose-related cytotoxic response at high levels of exposure. Furthermore, arsenic trioxide modulated caspase 3 activity and induced p38 MAP kinase activation in A549 cells. However, cell-cycle studies showed no statistically significant differences in DNA content at subG1 check point between control and arsenic trioxide treated cells.

## 1. Introduction

Arsenic compounds have been use in Chinese and Western medicine for many years. Arsenic compounds have been used to treat a variety of ailments such as syphilis, yaws, amoebic dysentery and trypanosomiasis [1]. Despite all of the adverse health effects associated with inorganic arsenic exposure, there is some evidence that small amounts of arsenic in the normal diet (10–50 ppb) may be beneficial to health [1]. Arsenic trioxide has been used as a mitochondria-targeting drug in acute promyelocytic leukemia [2–5]. Arsenic trioxide and arsenic containing compounds are cytotoxic [4–6] and capable of triggering apoptosis [6–10]. Their cytotoxic potential has been linked to a decreased mitochondrial potential, fragmented DNA, and finally apoptotic cell death [1,6,8,11]. Cellular proliferation or DNA synthesis is controlled by the cell cycle. Biological, pathophysiological and environmental factors can affect the cell cycle progression. It implies that cell numbers can be regulated by factors that influence cell survival as well as those that control proliferation, apoptosis and differentiation [12].

Apoptosis is an active and gene directed programmed cell death. Its role is to maintain tissue homeostasis and to eliminate excess or dysfunctional cells. Apoptosis is characterized by several morphological changes which include cell shrinkage, membrane blebbing, chromatin condensation, and nuclear fragmentation [13]. It is also characterized by biochemical features such as caspase cascade, and cleavage of various caspase substrates (caspase 3 and 9 activation) [13,14]. In apoptosis, the functional integrity of the plasma membrane is long maintained. In addition, apoptosis is accompanied by internucleosomal DNA fragmentation giving rise to the classical “ladder” pattern on DNA electrophoresis [4,12,15].

Arsenic, in particular, arsenic trioxide can also exert its effect through the activation of the mitogen-activated protein kinase (MAPK) cascade [16]. Environmental stress signals (ultraviolet light, heat or synthesis inhibitors) have been shown to activate the c-jun NH2- terminal kinase (JNK) and p38 MAPK pathways, which resulted in stress responses, growth arrest and/or apoptosis [16]. Verma *et al.* were the first to provide evidence that arsenic trioxide activated α and β isoforms of p38 MAP kinase in acute promyelocytic leukemia (NB-4) and in an all-*trans*-retinoic acid resistant NB-4 variant [17].

The aims of this study were to evaluate the effects of arsenic trioxide on DNA synthesis and to determine whether arsenic–induced apoptosis is mediated via caspase activation, p38 mitogen-activated protein kinase (MAPK), and cell cycle arrest.

## 2. Materials and Methods

### Cell Lines and Chemicals

Human lung carcinoma cell line (A549) and F-12 K medium were purchased from American Type Culture Collection (ATCC) (Manassa, VA). Fetal bovine serum (FBS), penicillin/streptomycin/fungizone, phosphate buffered saline (PBS) and trypsin versene were purchased from Invitrogen (Grand Island, NY). [^3^H methyl]Thymidine was obtained from MP Biomedicals (Irvine, CA). Arsenic trioxide (As_2_O_3_; lot # 02765-24) was purchased from Fisher Scientific (Houston, TX). Caspase-3 FITC kit was obtained from EMB Biosciences (San, Diego, CA) and the p38 MAP Kinase assay kit (non-radioactive) from Cell Signaling Technology, Inc (Danvers, MA).

### Cell Culture

A549 cells were maintained in F-12K complete growth medium (10% FBS, and 1% penicillin (10,000 units/mL) and streptomycin (10,000 units/mL) as adherent cells. The cells were grown in a humidified incubator under an atmosphere of 95% air and 5% CO_2_ at 37 ºC to sub-confluence (80–95%). After growing to 80–95% confluence, the medium was aspirated off and the cell monolayer was washed three times with sterile phosphate buffered saline (PBS). The cell monolayer was treated with 1 mL trypsin versene per plate and incubated briefly at 37 ºC. The cells were then viewed microscopically to ensure a complete cell detachment. Cells were re-suspended in F-12K complete medium and seeded in 13 × 100 mm tissue culture plates at a density of 5 × 10^5^ cells/plate, prior to arsenic trioxide treatment.

### [^3^H]Thymidine Incorporation Assay

Cells were seeded in 6 well culture plates at a density of 3 × 10^5^ cells/well and the cells grew to 60%–70% confluent in 3 days. Sub-confluent cells were incubated in serum starved medium (1% fetal bovine serum and 1% penicillin (10,000 units/mL)/streptomycin (10,000 units/mL) overnight, and then treated with different doses (0, 2, 4, 6, 8, 10 μg/mL) of arsenic trioxide. Cells were incubated for 18 h before 1μ Ci/mL [^3^H methyl]thymidine was added to each well for an additional 6 h period. The incubation of tritiated thymidine was terminated by aspirating the culture medium off, washed 3 times with cold phosphate buffered saline pH 7.4 followed by the addition of 2 ml/well of ice-cold 10% trichloroacetic acid (TCA) for 20 min at 4 ºC. The TCA was aspirated off the cells and the cells were washed three times with ice-cold water. After the washing, the cells were solubilized with 1 mL of 0.5 M NaOH/well at 37 ºC for 30 min. The [^3^H]thymidine incorporation was quantitated in a scintillation counter (Beckman Instruments). Each treatment group was assayed in triplicates and each experiment was repeated at least three times.

### Caspase-3 FITC Assay

Cells were seeded in 6 well tissue culture plates at a density of 3 × 10^5^ cells/well and grown to 60%–70% confluence for 3 days. Sub-confluent cells were placed in serum starved medium (1% fetal bovine serum and 1% penicillin (10,000 units/mL)/streptomycin (10,000 units/mL) overnight, and then treated with arsenic trioxide at 0, 2, 4, 6 μg/mL, respectively for 48 h. The caspase 3-FITC assay was performed using the manufacture’s recommended protocol. Caspase-3 activity was measured using a fluorescence-activated cell-sorting (FACS Vantage) system (Becton-Dickinson, San Jose, CA).

### Cell Cycle Analysis

Cell cycle analysis was performed as described by Ndebele *et al.* [18–20]. Briefly, A549 cells were seeded on 13 × 100 mm plates at a density of 3 × 10^5^/ plate until 75% confluent. Cells were incubated overnight in complete growth medium supplemented with 1% fetal bovine serum. Cells were treated with arsenic trioxide at 0, 2, 4, and 6μg/mL for 48 h. After the 48 h incubation, the cells were washed with cold PBS, harvested and counted. The cells density of 1 × 10^6^ was spun down at 3000 rpm for 5 min. The pellet was resuspended in 250 μL of propidium iodide solution. The reaction was incubated at 4 ºC in the dark for 1 h. The cell cycle distribution was measured using FACS Vantage flow cytometry system. This method allows for the calculation of the percentages of cells in the G0/G1 (resting phase), S (DNA synthesis) and G2M and apoptotic fractions (sub G1).

### P38 MAP Kinase Assay

549 cells were treated with arsenic trioxide at 0, 2, 4, and 6 μg/mL for 48 h and subsequently lysed and immunoprecipitated with immobilized phosphor-p38 MAPK (Thr 180/Tyr 182) mAb primary antibody. The slurry was incubated with gentle rocking overnight at 4 ºC. The immunoprecipitates were then washed two times in cell lysis buffer [20 mM Tris (pH 7.5), 150 mM NaCl, 1 mM EDTA, 1 mM EGTA, 1% Triton, 2.5 mM NaVO_4_, 1 μg/mL leupeptin] followed by two washes with kinase buffer [25 mM Tris (pH7.4), 5 mM β-glycerolphosphate, 2 mM DTT, 0.1 mM NaVO_4_, 10 mM MgCl_2_]. The immunoprecipitates were resuspended in 50 μL of kinase assay buffer with 200 μM ATP and kinase substrate (ATF-2 fusion protein). The reaction was incubated for 30 min at 30ºC and terminated with 50 μL of 3X sodium dodecylsulfate (SDS) sample buffer. Protein was analyzed by SDS-PAGE and ATF-2 as a substrate was assayed by western blotting and chemiluminescence detection.

### Statistical Analysis

Date collected from [^3^H]thymidine, caspase-3 and cell cycle distribution assays were represented as means ± SEs of three experiments performed in triplicates. They were analyzed by ANOVA using Microscoft Windows Excel statistical program, followed by the Student t- test to assess if there were any significant differences in mean values between control and each of the ATO treated cells. The statistical significance was considered at p < 0.05.

## 3. Results

### [^3^H]Thymidine Incorporation Assay

[^3^H]Thymidine incorporation assay was used to assess DNA synthesis in A549 cells. Data obtained from this assay showed a dose dependent effect of arsenic trioxide exposure (Figure 1). DNA synthesis decreased as arsenic trioxide concentration increased. The percentages of DNA synthesis were 100 ± 5, 88 ± 5, 64 ± 3, 13 ± 3, 0.02 ± 0.03, and 0.02 ± 0.02 for 0, 2, 4, 6, 8, and 10 μg/mL arsenic trioxide, respectively.

### Caspase 3-FITC Analysis

Data from Figure 2 indicates that caspase-3 levels were 0.074 ± 0.019, 0.19 ± 0.0, 0.46 ± 0.014, 1.02 ± 0.25 for 0, 2, 4, and 6 μg/mL, respectively. Statistically significant differences (p < 0.05) in caspase-3 activity were observed at 4 and 6 μg/mL arsenic trioxide compared to the control.

### Effect of Arsenic Trioxide on p38 MAPK Activity

The p38 MAPK activity was measured in the immunoprecipitate with phosphor p38 MAPK mab primary antibody in the presence of ATP and GST-ATF-2 fusion protein as substrate. Results showed a high expression of p38 MAPK (ATF-2) at 4 μg/mL of arsenic trioxide when compared to the control (0 μg/mL) (Figure 3).

### Effect of Arsenic Trioxide on Cell Cycle Distribution

The histograms in Figure 4 show the cell cycle distribution of A549 cells treated with arsenic trioxide at 0 μg/mL, 2 μg/mL, 4 μg/mL and 6 μg/mL for 48 h. The apoptotic fraction, the sub-G1 phase of the cell cycle, is represented by the M1 peak on the histograms. The other phases of the cell cycle (S, G1 and G2) are represented on the histograms as M2, M3 and M4, respectively.

The percentages of cells undergoing apoptosis represented by subG1 (M1) were 2.4%, 6.3%, 2.2% and 0.97% for 2, 4, and 6 μg/mL for 0, 2, 4, and 6 μg/mL, respectively.

DNA flow cytometric analysis indicated that treatment of arsenic trioxide (0–6 μg/mL) did not significantly (p < 0.05) alter the cell cycle distribution (Figure 5).

The graph represents the sub-G1 (M1), G1/G0 (M2), S (M3) and G2 (M4) percentages of cell cycle distribution from three independent experiments performed in triplicates.

## 4. Discussion

[^3^H]thymidine incorporation is a tool used by many researchers to evaluate the proliferative response to a chemical or drug. The [^3^H]thymidine is incorporated in the DNA to determine the percentage of cells in the DNA synthesis (S) phase of the cell cycle [21]. In this study, we demonstrated that arsenic trioxide inhibited DNA synthesis in a dose-dependent manner in lung adenocarcinoma cell line (A549). DNA synthesis significantly decreased at the highest level of exposure. Tchounwou *et al.* revealed a dose-response in human liver carcinoma cells (HepG2) and human leukemia cells (HL-60) in reference to arsenic toxicity [7,22]. Chow *et al.* reported that the arsenic trioxide (0.25–2 μM) exhibited inhibitory effects on the proliferation of MCF-7 cells in a dose and time dependent manner [23]. Lau *et al.* confirmed that cell proliferation occurred at a lower level of arsenite (2 μM) in rat LEC cell line [24]. In our study, we have shown that DNA synthesis was inhibited by arsenic trioxide in A549 cells. Chow *et al.* also demonstrated that arsenic was capable of reducing cell survival in MCF-7 cells via the suppression of the estrogen-induced growth stimulatory effects in MCF-7 cells [22]. Li *et al.* tested the effects of arsenic trioxide in human pancreatic (AsPC-1), colonic (HT-29), and breast (MCF-7) cancer cells [25]. Their study revealed that in all three cancer cell lines, arsenic trioxide inhibited proliferation in a concentration and time-dependent manner, as measured by [^3^H-methyl]thymidine incorporation and cell counting [25]. In comparison, studies performed by Harnagea-Theophilis *et al.* revealed that DNA synthesis was not stimulated in estrogen-responsive breast cells (MCF-7) when combined with estradiol and acetaminophen [21].

Apoptosis is an active and gene-directed form of cell death with well characterized morphological and biochemical features, including activation of a caspase cascade, DNA fragmentation into nucleosomal fragments, and cleavage of various caspase substrates [4,13,15]. Studies have shown that arsenic induces apoptosis in several cell lines [9–11,25–27]. In our study, we demonstrated that arsenic trioxide also induces apoptosis in lung carcinoma (A549) cells especially at higher levels of exposure. Previous studies have revealed that arsenic trioxide could induce apoptosis at both lower and higher levels of exposure in renal, myelocytic, and liver cancer cells [25].

According to Hockenberry *et al.*, the “ladder” pattern or DNA fragmentation is the indication of apoptosis [15]. Previous work in our laboratory has shown the laddering pattern in A549 cells after 48 h treatment of arsenic trioxide at 2 μg/mL, 4 μg/mL and 6 μg/mL [28]. Coincidentally, with inhibition of growth, Li *et al.* observed other morphological changes in the colon, breast and pancreatic cancer cell lines after exposure to arsenic trioxide. These changes included reduced cytoplasmic volume, membrane blebbing, and nuclear condensation, consistent with apoptosis [26]. Zheng *et al.* showed that arsenic trioxide induced apoptosis in human papilloma viruses (HPV16) DNA- immortalized human cervical epithelial cells *in vitro* and selectively inhibited early viral gene expression [28].

Caspases are a family of proteases that play a pivotal role in the execution of apoptosis [29]. Caspase-3 was used as a biomarker for apoptosis in this study. We observed that caspase 3 activity increased significantly at 6 μg/mL (3μM) of arsenic trioxide compared to the control. Previous studies have shown that caspase 3 plays an essential role as an executor in apoptosis [30,31]. Han and Kim *et al.* observed a significant increase of caspase-3 activity in juxtaglomerular cells (As4.1 JG) treated with 7 μM arsenic trioxide for 48 h compared to control [31]. They incubated the arsenic-treated As4.1 JG cells with caspase 3 inhibitor. The susceptibility of arsenic trioxide in A549 cells is lower than that in renal cells. The differing susceptibilities might result from the origin of the cells.

One of the mechanisms by which arsenic exerted its effects was through signal-transduction pathways. Therefore, we studied the effect of arsenic trioxide on p38 MAP kinase in regards to apoptosis. Lau *et al.* conducted research on arsenic and its effect on signal transduction and found that arsenic altered the signal transduction via MAP kinase [23]. In our study, we observed an up–regulation p38 MAPK (ATF-2 substrate expression) at 4 μg/mL of arsenic trioxide. Namgung and Xia have reported that the neurotoxicity of arsenite may cause the activation of p38 and c-Jun N- terminal kinase 3 (JNK3) and mitogen-activated protein kinases (MAPKs), which are involved in the apoptosis process [6,32].

In our study, we observed little to no effect of arsenic on cell cycle distribution in the sub-G1 phase in A549 treated at 48 h. Han and Kim reported that arsenic trioxide did not significantly alter the cell cycle distribution in A549 cell treated for 72 h. However, they pointed out that 50 μM arsenic trioxide induced a G2 arrest in these same cells [33]. In a previous study, they reported that low doses of arsenic trioxide (1–10 μM) in As4.1 JG cells induced S phase arrest [31]. In the same cell line, they revealed that 1 μM arsenic trioxide did not trigger apoptosis. However, they reported a 50% elevation in the percentages of sub-G1 population in As4.1 JG at 20 μM arsenic trioxide [31]. We observed that arsenic trioxide induces a G0/G1 phase arrest in a dose-dependent manner. The G0/G1 phase percentages increased by two-fold at 6 μg/mL exposure. Liu and Hilsenbeck reported that arsenic trioxide-induced apoptosis and p53-dependent G_1_ or G_2_/M cell cycle arrest in multiple myeloma cells [34]. They stated that the multiple myeloma cells with normal p53 were resistant to arsenic trioxide-induced apoptosis and were arrested in G1 phase, whereas cells lacking functional p53 were sensitive and were arrested in G2/M phase [33]. Li and others revealed the redistribution of the cell cycle in solid human tumor such as breast, colon and pancreas [24].

## 5. Conclusions

Arsenic is a well documented human carcinogen associated with various cancers, including lung cancer. Trisenox is a drug for treating myeloma cancer, but little is known about its mechanism in treating solid tumors such as non-small cell tumor lung. We have demonstrated that arsenic trioxide inhibited DNA synthesis in a dose-dependent manner in A549 cells. Our findings also showed that arsenic trioxide induces apoptosis via caspase-3 activation and influenced the p38 MAP kinase pathway. Although, arsenic trioxide did not alter the subG1 of the cell cycle, it significantly modulated the G0/G1 phase. The susceptibility of arsenic trioxide on sub-G0 and G0/G1 appears to depend on the origin of the cell (cell type). These results will lead to a better understanding of the role played by arsenic trioxide-induced apoptosis in the treatment of lung cancer.

## Figures and Tables

**Figure 1.**
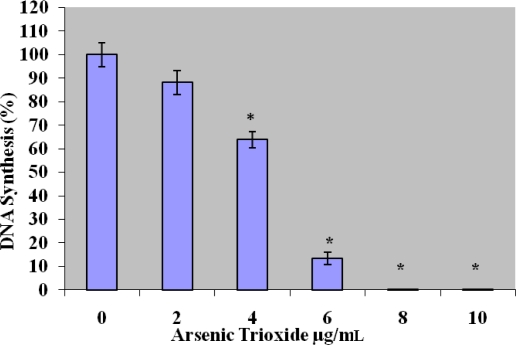
[^3^H]thymidine incorporation assay of A549 cells after 48 h exposure to arsenic trioxide. The data is represented as mean ± SEM of three experiments performed in triplicates. The differences were considered statistically significant with a p value < 0.05. The significance of the value is indicated by asterisks (*).

**Figure 2.**
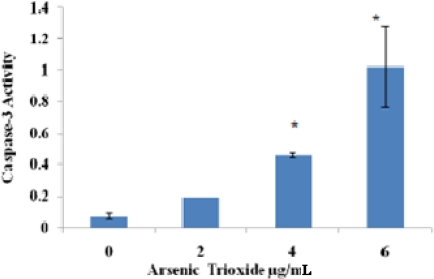
Effect of arsenic trioxide on caspase-3 activity in A549 cells.

**Figure 3a.**
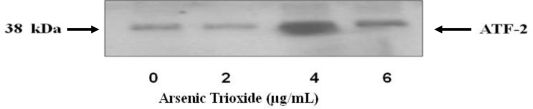
Effect of arsenic trioxide on p38 MAPK expression in A549 cells.

**Figure 3b.**
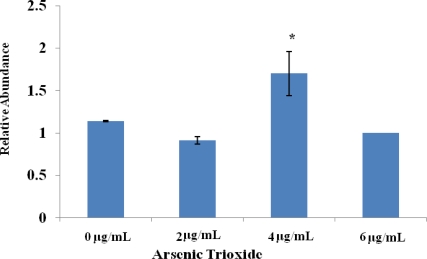
Densiometric analysis of p38 MAPK expression in A549 cells exposed to arsenic trioxide. Data represent the mean values of three individual experiments done in triplicates. The intensities of the bands represented in Figure 3a were quantified and plotted as relative abundances.

**Figure 4.**
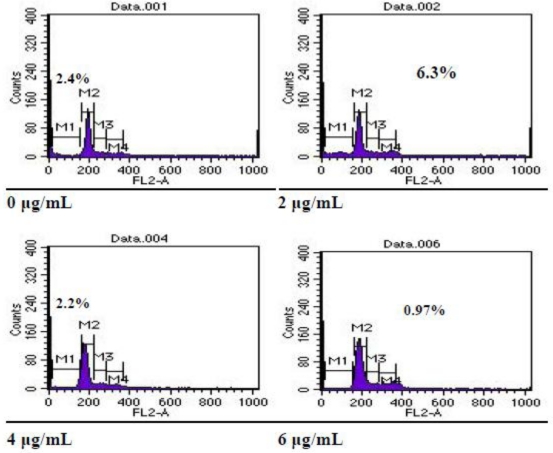
Representative cell cycle histograms of A549 cells treated with arsenic trioxide (0, 2, 4, and 6 μg/mL) for 48 h.

**Figure 5.**
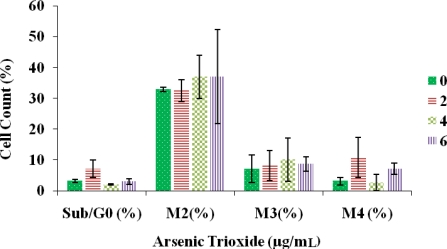
Effect of arsenic trioxide on cell cycle distribution in A549 cells.
